# Regulation of nerve-evoked contractions of rabbit vas deferens by acetylcholine

**DOI:** 10.14814/phy2.12520

**Published:** 2015-09-09

**Authors:** Audrey Wallace, Deborah Gabriel, Noel G McHale, Mark A Hollywood, Keith D Thornbury, Gerard P Sergeant

**Affiliations:** 1 The Smooth Muscle Research Centre, Dundalk Institute of TechnologyDundalk, Co. Louth, Ireland

**Keywords:** Cholinergic, muscarinic receptors, smooth muscle, vas deferens

## Abstract

Stimulation of intramural nerves in the vas deferens of many species yields a classical biphasic contraction comprised of an initial fast component, mediated by P_2_X receptors and a second slower component, mediated by *α*_1_-adrenoceptors. It is also recognized that sympathetic nerve-mediated contractions of the vas deferens can be modulated by acetylcholine (Ach), however there is considerable disagreement in the literature regarding the precise contribution of cholinergic nerves to contraction of the vas deferens. In this study we examined the effect of cholinergic modulators on electric field stimulation (EFS)-evoked contractions of rabbit vas deferens and on cytosolic Ca^2+^ levels in isolated vas deferens smooth muscle cells (VDSMC). The sustained component of EFS-evoked contractions was inhibited by atropine and by the selective M_3_R antagonist, 1,1-dimethyl-4-diphenylacetoxypiperidinium iodide (4-DAMP). EFS-evoked contractions were potentiated by Ach, carbachol (Cch), and neostigmine. The sustained phase of the EFS-evoked contraction was inhibited by prazosin, an *α*_1_-adrenoceptor antagonist and guanethidine, an inhibitor of noradrenaline release, even in the continued presence of Ach, Cch or neostigmine. The soluble guanylate cyclase (sGC) inhibitor, 1H-[1,2,4]Oxadiazolo[4,3-a]quinoxalin-1-one enhanced the amplitude of EFS-evoked contractions and reduced the inhibitory effects of 4-DAMP. Isolated VDSMC displayed spontaneous Ca^2+^ oscillations, but did not respond to Cch. However, the *α*_1_-adrenoceptor agonist, phenylephrine, evoked a Ca^2+^ transient and contracted the cells. These data suggest that EFS-evoked contractions of the rabbit vas deferens are potentiated by activation of M_3_ receptors and reduced by activation of a sGC-dependent inhibitory pathway.

## Introduction

Contraction of the vas deferens is responsible for the movement of sperm from the epididymis to the urethra and its dysfunction is associated with the occurrence of several ejaculatory disorders, including premature ejaculation (Michel 2007; Burnstock 2009; Buvat 2011). Peristaltic contractions of the smooth muscle cells that surround the vas deferens are activated by sympathetic neurons, which have numerous varicosities that release adenosine trisphosphate (ATP) and noradrenaline (NA) to initiate contraction (Westfall et al. 1978). Stimulation of sympathetic nerves in the vas deferens leads to a classical biphasic contractile response, composed of an initial transient contraction, sometimes referred to as a “twitch” contraction followed by a secondary sustained contraction, known as the “hump” contraction. The transient response is brought about by activation of postjunctional P_2_X receptors by ATP, whereas the secondary component involves activation of postjunctional *α*_1_-adrenoceptors by NA (Ventura 1998; Burnstock and Verkhratsky 2010; Koslov and Andersson 2013).

The vas deferens also contains a rich population of cholinergic nerves (Furness and Iwayama 1972; Gosling and Dixon 1972; Majcen 1984), although their precise role and contribution to the biphasic contractions described above remains unclear (Koslov and Andersson 2013). This is partly due to conflicting reports in the literature. For example, some studies showed that electric field stimulation (EFS)-evoked contractions of the vas deferens were inhibited by the muscarinic receptor (MR) antagonist atropine (Fukushi and Wakui 1986; White et al. 2010) whereas as others showed that atropine had little, or no effect (Nakanishi et al. 2004; Cuprian et al. 2005). Similarly, Canevari et al. (1986) reported that exogenous application of cholinergic drugs to human vas deferens were totally inactive, whereas Miranda et al. (1992) found that exogenous application of acetylcholine (Ach)-induced concentration-dependent contractions.

Other studies have reported a modulatory role for Ach in the vas deferens rather than a direct contribution to the EFS-evoked contraction. However, while (Lee 1985; Miranda and Wolstenholme 1985; Matsuno and Mita 1992) reported that carbachol (Cch) and Ach potentiated contractions of mouse, rat, and guinea-pig vas deferens, respectively, (Eltze 1988; Eltze et al. 1988; Grimm et al. 1994) showed that Cch reduced EFS-evoked contractions of rabbit vas deferens. Therefore, the precise role of Ach in the vas deferens is still unclear and requires further investigation. This is important, especially as recent studies have advocated pharmacological inhibition of the autonomic pathways that regulate contraction of the vas deferens as a method to inhibit sperm transport and develop a male contraceptive (White et al. 2013).

## Methods

### Animal welfare and ethical statement

All experiments were approved by the Dundalk Institute of Technology (DkIT) Animal Care and Use committee and were in accordance with EU Directive 2010/63/EU. New Zealand White rabbits (16–20 weeks old) were humanely killed with a lethal injection of pentobarbitone (120 mg kg^−1^, i.v.). The vasa deferentia were removed and placed in Krebs’ solution for further use.

### Tension recordings

Longitudinal segments of vas deferens (˜8 mm in length) were dissected from the mid section of the tissue. These were mounted in water-jacketed organ baths maintained at 37°C and bathed with Krebs’ solution bubbled with 95% O_2_–5% CO_2_. Tissue segments were adjusted to a tension of 2–4 mN and allowed to equilibrate for 50 min before experimentation began. Contractions were recorded using the multichannel Myobath system and data were acquired using DataTrax2 software (World Precision Instruments, Hertfordshire, U.K.). EFS was applied via two platinum electrode wires (5 mm length, 2.5 mm apart) by a MultiStim system-D330 stimulator (Digitimer Ltd, Hertfordshire, U.K.), which delivered 0.3-ms pulses of 20 V (nominal) at a frequency of 4 Hz for 1 min.

### Calcium imaging

Vas deferens segments were cut into 1 mm^3^ pieces and stored in Ca^2+^-free Hanks solution for 30 min prior to cell dispersal. Tissue pieces were incubated in dispersal medium containing (per 5 mL) of Ca^2+^-free Hanks solution (see solutions): 15 mg collagenase (Sigma type 1A), 1 mg proteinase (Sigma type XXIV), 10 mg bovine serum albumin (Sigma, Wicklow, Ireland), and 10 mg trypsin inhibitor (Sigma) for 10–15 min at 37°C. Tissue was then transferred to Ca^2+^-free Hanks solution and stirred for a further 15–30 min to release single smooth muscle cells. These were plated in Petri dishes containing 100 *μ*mol/L Ca^2+^ Hank's solution and allowed to settle in glass bottomed Petri dishes until they had stuck down. They were then incubated in 0.4 *μ*mol/L fluo-4/AM (Molecular Probes, Dublin, Ireland) for 6–8 min in the dark at room temperature before being studied. During experiments, the dish containing the cells was continuously perfused with Hanks solution at 36 ± 1°C. Additionally, the cell under study was continuously superfused by means of a custom built close delivery system with a pipette of tip diameter 200 *μ*m placed ˜300 *μ*m from the cell. The Hanks solution in the close delivery system could be switched to a drug-containing solution with a dead-space time of less than 5 sec.

Cells were imaged using an iXon 887 EMCCD camera (Andor Technology, Belfast, Ireland; 512 × 512 pixels, pixel size 16 × 16 *μ*m) coupled to a Nipkow spinning disk confocal head (CSU22; Yokogawa, Tokyo, Japan). A krypton-argon laser (Melles Griot, Rochester, NY) at 488 nm was used to excite the fluo-4, and the emitted light was detected at wavelengths >510 nm. Experiments were performed using a 60× objective (Olympus, Southend-on-Sea, U.K.) resulting in images of pixel size 0.266 × 0.266 *μ*m. Images were acquired at 15 frames per second. Background fluorescence from the camera, obtained using a null frame, was subtracted from each frame to obtain “F.” F_0_ was determined as the minimum fluorescence measured between oscillations under control conditions. To obtain post hoc pseudo line-scan images for display in figures, a one pixel thick line was drawn centrally through the entire length of the cell and the “reslice” command in Image J (NIH, Bethesda, MD) was invoked. Plots of F/F_0_ were obtained from the post hoc line-scan by drawing a rectangle around the entire area of the line-scan image and plotting the intensity profile in Image J. _Δ_F/F_0_ refers to the measurement of the change in Ca^2+^ levels from basal to peak.

### Materials

The solutions used were of the following composition (mmol/L): Normal Hanks Solution: NaCl (125.0), KCl (5.4), Glucose (10.0), Sucrose (2.9), NaHCO_3_ (4.2), KH_2_PO_4_ (0.4), NaH_2_PO_4_ (0.3), MgCl_2_·6H_2_O (0.5), CaCl_2_·2H_2_O (1.8), MgSO_4_ (0.4), Hepes (10.0). pH to 7.4 using NaOH. pH adjusted to 7.4 with NaOH; Ca^2+^*-*free Hanks solution (for cell dispersal): NaCl (125), KCl (5.36), glucose (10), sucrose (2.9), NaHCO_3_ (15.5), KH_2_PO_4_ (0.44), Na_2_HPO_4_ (0.33), N-[2-Hydroxyethylpiperazine]-N′-[2-ethanesulfonic acid] (HEPES; 10) pH adjusted to 7.4 with NaOH. Krebs’ solution: NaCl (120), KCl (5.9), NaHCO_3_ (1.2), glucose (5.5) CaCl_2_ (2.5), MgCl_2_ (1.2) pH maintained at 7.4 by bubbling with 95% O_2_–5% CO_2_.

#### Drugs used

Atropine, 1,1-dimethyl-4-diphenylacetoxypiperidinium iodide (4-DAMP), 1H-[1,2,4]Oxadiazolo[4,3-a]quinoxalin-1-one (ODQ), Prazosin, Guanethidine, Neostigmine, Ach, Cch, *α*,*β*-methylene ATP, tetrodotoxin (TTX), and phenylephrine (PE). All drugs were purchased from Sigma, except 4-DAMP and ODQ which were supplied by Tocris and TTX which was supplied from Abcam Biochemicals (Cambridge, U.K.). Drugs were prepared as concentrated stock solutions in the appropriate solvent before being added to the tissue bath or reservoir to achieve the stated final concentration.

### Data analysis and statistical procedures

Summary data are presented as the mean ± SEM and statistical differences in experiments were compared using Student's paired *t*-test (two tailed test) taking the *P* < 0.05 level as significant. Multiple comparisons were analyzed using one-way analysis of variance (ANOVA), with a Bonferroni post hoc test. Throughout, “*n*” refers to the number of tissue segments or cells in each experimental series. These were obtained from a minimum of three animals.

## Results

**Figure 1:**
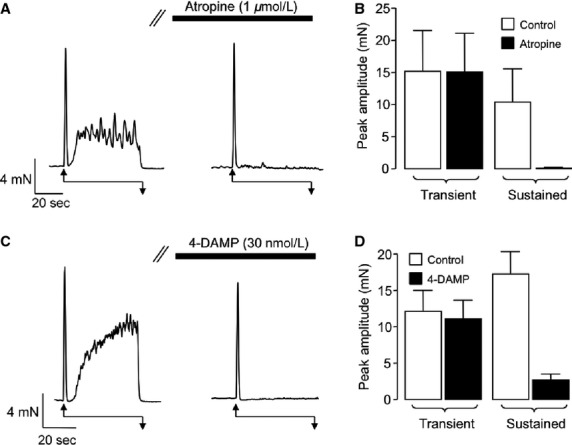
Effect of muscarinic receptor antagonists on EFS-evoked contractions of rabbit vas deferens. (A) Representative trace showing an EFS-evoked contraction before and during the presence of atropine (1 *μ*mol/L). Double arrows represent period of EFS in each example. (B) Summary plot showing mean, peak amplitude of transient, and sustained components of contraction before (control, open bar) and during atropine (filled bar). (C) Representative trace showing an EFS-evoked contraction before and during the presence of 4-DAMP (30 nmol/L). (D) Summary plot showing mean, peak amplitude of transient, and sustained components of contraction before (control, open bars) and during 4-DAMP (filled bars). EFS, electric field stimulation; 4-DAMP, 1,1-dimethyl-4-diphenylacetoxypiperidinium iodide.

EFS of isolated rabbit vas deferens segments yielded biphasic contractile responses that were reproducible when repeated at 20-min intervals. Both the transient and the sustained phase of the contraction were inhibited by TTX (1 *μ*mol/L), from 16.8 ± 1.8 and 10.5 ± 2.2 mN under control conditions to 0.28 ± 0.28 and 0.19 ± 0.17 mN, respectively (*n* = 5, *P* < 0.05). To test if the EFS-evoked contractions involved activation of muscarinic acetylcholine receptors, the effect of the nonselective MR antagonist, atropine (1 *μ*mol/L) was examined. Figure 1A is a representative trace showing responses to EFS before and during the presence of atropine. Atropine had little effect on the transient component, but abolished the sustained, second component of the contraction. This effect was consistent in six preparations where the mean amplitude of the second component of contraction was reduced from 10.4 ± 5.1 to 0.14 ± 0.04 mN (*P* < 0.05) and the transient contraction was not affected by the drug (15.24 ± 6.3 mN under control conditions vs. 15.15 ± 5.9 mN in atropine, *P* > 0.05). These summary data are plotted in Figure 1B. The representative trace and summary plots shown in Figure 1C and D, respectively, indicate that similar results were achieved with the selective M_3_R antagonist 4-DAMP (30 nmol/L). Thus, the sustained contraction was greatly reduced, from 17.3 ± 2.9 to 2.8 ± 0.7 mN in 4-DAMP (*n* = 8, *P* < 0.05), but the transient contraction was not significantly reduced (12.2 ± 2.8 mN under control conditions vs. 11.2 ± 2.5 mN in 4-DAMP, *P* > 0.05).

**Figure 2:**
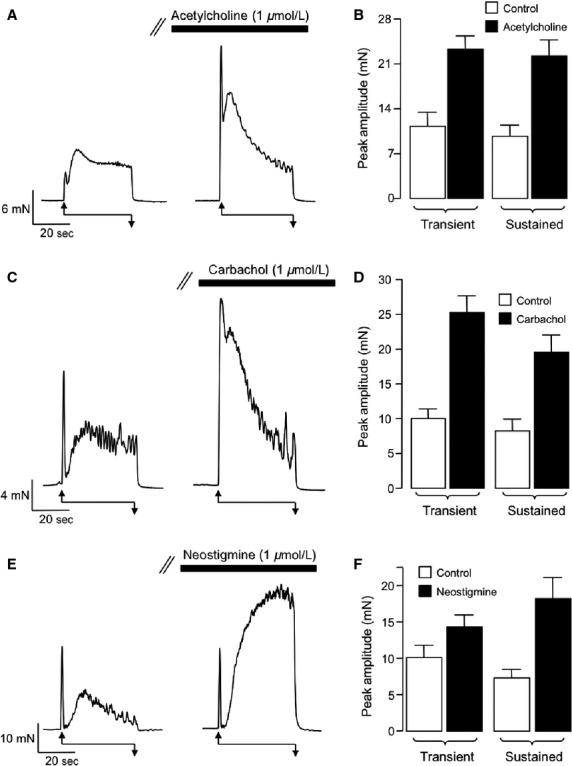
Effect of acetylcholine, carbachol, and neostigmine on electric field stimulation (EFS)-induced contractions. (A) Representative trace showing an EFS-evoked contraction before and during the presence of acetylcholine (1 *μ*mol/L). (B) Summary plot showing mean, peak amplitude of transient, and sustained components of contraction before (control, open bar) and during acetlcholine (filled bar). (C) Representative trace showing an EFS-evoked contraction before and during the presence of carbachol (1 *μ*mol/L). (D) Summary plot showing mean, peak amplitude of transient, and sustained components of contraction before (control, open bars) and during carbachol (filled bars). (E) Representative trace showing that the acetylcholinesterase inhibitor, neostigmine enhanced the sustained component of EFS-induced contractions. (F) Summary plot showing mean, peak amplitude of transient, and sustained components of contraction before (control, open bars) and during neostigmine (filled bars).

**Figure 3:**
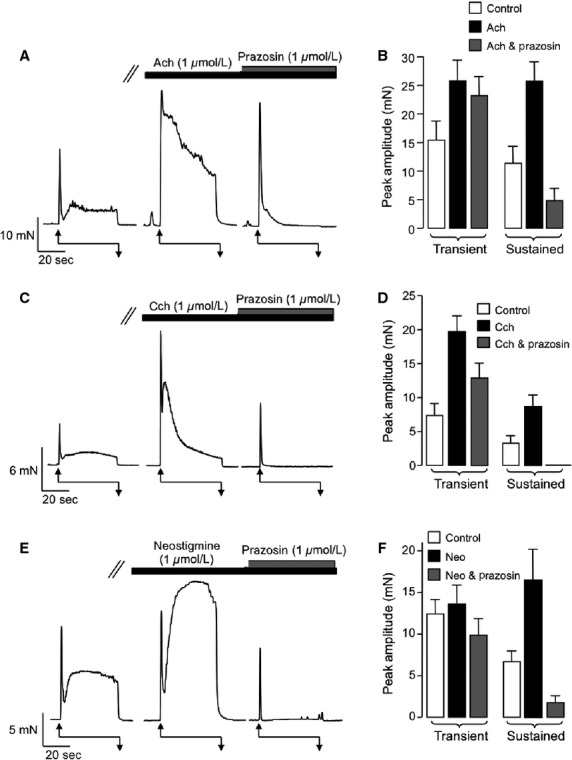
Prazosin inhibits the sustained component of electric field stimulation (EFS)-induced contractions. (A and B) Representative trace (A) and corresponding summary plot (B) showing representative and mean amplitude of peak EFS-induced contractions (1) before, (2) during the presence of acetylcholine (1 *μ*mol/L), and (3) during the presence of acetylcholine and prazosin (1 *μ*mol/L). (C and D) Representative trace (A) and corresponding summary plot (B) showing representative and mean amplitude of peak EFS-induced contractions (1) before, (2) during the presence of carbachol (1 *μ*mol/L), and (3) during the presence of carbachol and prazosin (1 *μ*mol/L). (E and F) Representative trace (A) and corresponding summary plot (B) showing representative and mean amplitude of peak EFS-induced contractions (1) before, (2) during the presence of neostigmine (1 *μ*mol/L), and (3) during the presence of neostigmine and prazosin (1 *μ*mol/L).

Next, we examined the effect of exogenous bath application of Ach (1 *μ*mol/L), the MR agonist Cch (1 *μ*mol/L) and the acetylcholinesterase inhibitor neostigmine (1 *μ*mol/L) on EFS-evoked contractions of vas deferens. Application of these agents did not evoke sustained tonic contractions. However, as can be seen from the respective representative records in Figure 2A, C, and E, Ach and Cch enhanced both components of each of EFS contraction, whereas neostigmine selectively enhanced the sustained component. Summary data for these effects are plotted in Figure 2B, D, and F, respectively. Each of these agents significantly enhanced the mean amplitude of the transient and sustained components of the EFS contractions (*n* = 19, *P* < 0.05), however, it was notable that neostigmine produced a smaller enhancement of the transient contraction compared to Ach or Cch. In order to test if these potentiation effects were due to direct stimulation of postjunctional Ach receptors, or indirectly, via release of NA acting on *α*_1_-adrenoceptors, we repeated the experiments, described in Figure 2, in the presence of the *α*_1_-adrenoceptor antagonist, prazosin (1 *μ*mol/L). Under control conditions application of prazosin abolished the sustained phase of contraction from a mean of 8.6 ± 2.5 mN (*n* = 5, *P* < 0.05) as previously demonstrated by Sneddon et al. (1984). The representative trace in Figure 3A and corresponding summary data in Figure 3B show that Ach significantly enhanced both transient and sustained phases of the contraction, but that the sustained component was significantly reduced in the presence of prazosin (*P* < 0.05, *n* = 7). Prazosin abolished the sustained phase of EFS contractions and also reduced the amplitude of the transient phase that was enhanced by Cch (Fig. 3C and D). However, the amplitude of the transient component was still significantly larger than under control conditions (*P* < 0.05, *n* = 8). Figure 3E and F show that neostigmine also enhanced the sustained contraction and that this response was dramatically reduced in the presence of prazosin (*P* < 0.05, *n* = 6). The residual transient contractions that remained in the presence of prazosin were abolished by application of the desensitizing P2X receptor agonist *α*,*β*-methylene ATP (1 *μ*mol/L, data not shown) suggesting that the transient component of the EFS contraction was mediated by P2X receptors. Taken together, these data suggest that potentiation of the second component of contraction by Ach, involves activation of *α*_1_-adrenoceptors, possibly by stimulating release of NA from sympathetic nerves. This idea was tested further by investigating if the stimulatory effects of Ach were affected by inhibition of NA release from sympathetic nerves using guanethidine.

**Figure 4:**
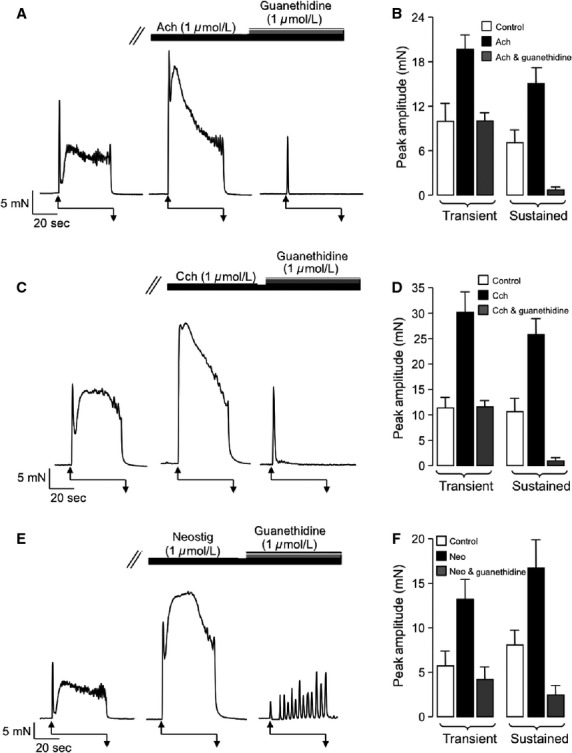
Guanethidine inhibits the sustained component of electric field stimulation (EFS)-induced contractions. (A and B) Representative trace (A) and corresponding summary plot (B) showing representative and mean amplitude of peak EFS-induced contractions (1) before, (2) during the presence of acetylcholine (1 *μ*mol/L), and iii) during the presence of acetylcholine and guanethidine (1 *μ*mol/L). (C and D) Representative trace (A) and corresponding summary plot (B) showing representative and mean amplitude of peak EFS-induced contractions (1) before, (2) during the presence of carbachol (1 *μ*mol/L), and (3) during the presence of carbachol and guanethidine (1 *μ*mol/L). (E and F) Representative trace (A) and corresponding summary plot (B) showing representative and mean amplitude of peak EFS-induced contractions (1) before, (2) during the presence of neostigmine (1 *μ*mol/L), and (3) during the presence of neostigmine and guanethidine (1 *μ*mol/L).

Figure 4A and B show the effect of guanethidine (1 *μ*mol/L) on EFS-induced contractions that were potentiated by Ach. Guanethidine significantly inhibited both components of contraction in the presence of Ach (*P* < 0.05, *n* = 7). However, the effect on the sustained element was notably greater than the transient contractions, which were not significantly different from those observed before addition of Ach (*P* > 0.05). Similar inhibitory effects of guanethidine were observed in response to stimulation by Cch (Fig. 4C and D, *n* = 9) and neostigmine (Fig. 4E and F, *n* = 9). These results further suggest that the excitatory effects of Ach were brought about by release of NA from sympathetic nerves.

**Figure 5:**
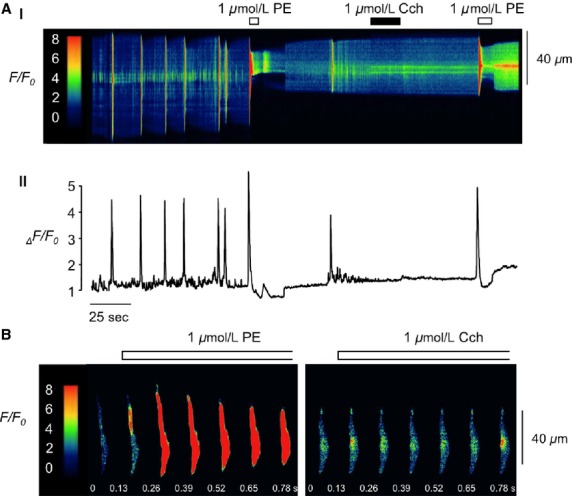
Effect of PE and Cch on Ca^2+^ levels in freshly isolated rabbit VDSMC. (A.i and A.ii) Representative pseudo line-scan image and corresponding intensity profile plot showing the effect of exogenous application of PE and Cch to a freshly isolated rabbit VDSMC. (B). Montage showing whole cell images of the effects of PE and Cch. VDSMC, vas deferens smooth muscle cells; PE, phenylephrine; Cch, carbachol.

The data above suggested that the stimulatory effects of Ach on EFS-evoked contractions were unlikely to be mediated by direct activation of postjunctional Ach receptors on vas deferens smooth muscle cells (VDSMC). To examine this issue more closely we examined the effect of Cch and the *α*_1_-adrenoceptor agonist, PE on Ca^2+^ levels in freshly isolated rabbit VDSMC. Figure 5A.i is a representative pseudo line-scan image that shows the effect of application of PE (1 *μ*mol/L) and Cch (1 *μ*mol/L) for 10 sec to the same cell and Figure 5A.ii is the corresponding fluorescence intensity profile plot. Under control conditions the cell exhibited a series of fast, localized Ca^2+^ oscillations, along with more intense global oscillations that occurred at a lower frequency and were associated with slight contractions of the cell. When PE was applied it caused an intense Ca^2+^ transient that induced a robust contraction of the cell. Following wash-out, the cell began to relax and the spontaneous activity returned. Next, Cch was applied, however, there was no discernable response to the drug. Finally, following a ˜50 sec interval, PE was reapplied to the cell, evoking a sharp rise in Ca^2+^, similar to the initial response. Figure 5B are montages, obtained from the record in Figure 5A, that compare the response of the cell to application of PE and Cch. These data are representative of six similar experiments whereby PE evoked a Ca^2+^ transient with a mean amplitude of 6.3 ± 1.1 _Δ_F/F_0_. Cch was without effect in every cell.

**Figure 6:**
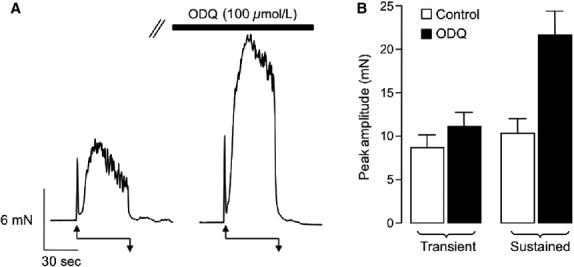
ODQ enhances the sustained component of EFS-induced contractions. (A) Representative trace showing an EFS-evoked contraction before and during the presence of the sGC inhibitor, ODQ (100 *μ*mol/L). (B) Summary plot showing mean, peak amplitude of transient, and sustained components of contraction before (control, open bar) and during ODQ (filled bar). EFS, electric field stimulation; sGC, soluble guanylate cyclase.

The data presented thus far suggest that Ach potentiates EFS-induced contractions of rabbit vas deferens by stimulating release of NA from sympathetic nerves. However, a puzzling aspect of the study was; why, in the presence of atropine and 4-DAMP, was there not a contractile response resulting from direct stimulation of sympathetic nerves? We speculated that one potential reason for this could be that EFS also activates an inhibitory pathway that opposes contraction, such that in the absence of the excitatory Ach effects, this inhibitory effect dominates. Studies by Gur et al. (2010) and da Silva et al. (2012) showed that EFS-evoked contractions of rat vas deferens were enhanced by the nitric oxide (NO) synthase inhibitor l-NAME, indicating an inhibitory role for NO in the rat vas deferens. NO is known to relax smooth muscle by activating the enzyme soluble guanylate cyclase (sGC). Therefore, we examined if a sGC-dependent mechanism affected EFS-evoked contractions in this study by examining the effect of ODQ, a sGC inhibitor, on EFS-induced contractions. Figure 6A shows a representative EFS-induced contraction, before, and during application of ODQ (100 *μ*mol/L). ODQ dramatically enhanced the sustained element of the contraction and this effect was repeatable in 16 preparations in which the mean amplitude of the sustained contraction increased from 10.4 ± 1.6 mN under control conditions to 21.7 ± 2.7 mN in ODQ, *n* = 16, *P* < 0.05). ODQ also produced a small, but significant enhancement of the transient contraction amplitude from 8.7 ± 1.4 mN before, to 11.2 ± 1.6 mN during the addition ODQ (*n* = 16, *P* < 0.05). These data suggest that the amplitude of EFS-evoked contractions in the vas deferens may be negatively regulated by activation of sGC.

**Figure 7:**
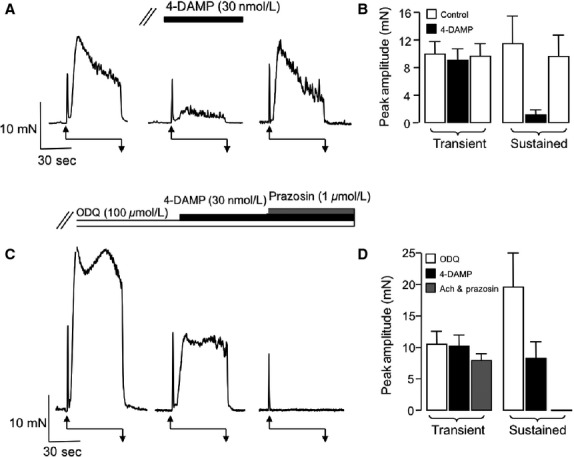
Effect of 4-DAMP on EFS-induced contractions, before, and during the presence of ODQ. (A) Representative trace showing EFS-evoked contractions before, during the presence of 4-DAMP, and following wash-out. (B) Summary plot showing mean, peak amplitude of transient, and sustained components of contraction in the absence (open bars) and presence of 4-DAMP (filled bar). (C) Continuation of representative trace shown in (A). ODQ enhanced EFS-induced contractions and the effect of 4-DAMP was diminished in the presence of 4-DAMP, compared to control. (D) Summary plot showing mean, peak amplitude of transient, and sustained components of contraction in ODQ (open bars) ODQ & 4-DAMP (filled bar) and ODQ, 4-DAMP and prazosin (gray bar). 4-DAMP, 1,1-dimethyl-4-diphenylacetoxypiperidinium iodide; EFS, electric field stimulation.

Lastly, we reasoned that, in the absence of the ODQ-sensitive inhibitory pathway, the inhibitory effects 4-DAMP should be diminished, as the effect of direct stimulation of sympathetic nerves should now be revealed. To test this hypothesis we examined the effect of 4-DAMP (30 nmol/L) on EFS-induced contractions on the same preparation, before, and during the presence of ODQ (100 *μ*mol/L). Figure 7A is a representative trace that shows a series of EFS-evoked contractions, before, during, and following wash-out of 4-DAMP (30 nmol/L). 4-DAMP selectively inhibited the second component of contraction and this observation is reflected in the summary plot in Figure 7B indicating that the sustained contraction was significantly reduced by ˜90% (from 11.5 ± 4 to 1.2 ± 0.7 mN, *P* < 0.05, *n* = 6). Following wash-out of 4-DAMP, the cell was exposed to ODQ, which enhanced the contractile response (Fig. 7C). 4-DAMP was then reapplied and its inhibitory effects were notably smaller compared to control. For example, in the presence of ODQ, 4-DAMP reduced the sustained contraction amplitude by ˜57% from 19.6 ± 5.4 to 8.3 ± 2.6 mN (*P* < 0.05, *n* = 6, Fig. 7D). Finally, prazosin was added to bath and the remaining sustained component of EFS-induced contraction was completely abolished.

## Discussion

This study examined the effect of Ach on EFS-evoked contractions of rabbit vas deferens. EFS of the rabbit vas deferens produces robust contractions that have the classical biphasic contraction, consisting of an initial transient and secondary sustained component (Sneddon et al. 1984). EFS-evoked contractions of the rabbit vas deferens are known to be modulated by Ach (Eltze 1988; Eltze et al. 1988; Grimm et al. 1994), therefore it is an appropriate model to examine the effect of Ach modulators on EFS-evoked contractions of the vas deferens. The main findings of this study were as follows: (1) the sustained phase of EFS-evoked contractions of rabbit vas deferens was abolished by atropine and the selective M_3_R antagonist 4-DAMP; (2) Contractions were potentiated by Ach, Cch, and neostigmine, and these responses were inhibited by prazosin, an *α*_1_-adrenoceptor antagonist and guanethidine, an inhibitor of NA release; (3) The sGC inhibitor, ODQ enhanced the amplitude of EFS-evoked contractions and reduced the inhibitory effects of 4-DAMP; (4) Isolated VDSMC displayed spontaneous Ca^2+^ oscillations, but did not respond to Cch. However, the *α*_1_-adrenoceptor agonist, PE evoked a Ca^2+^ transient and contracted the cells.

**Figure 8:**
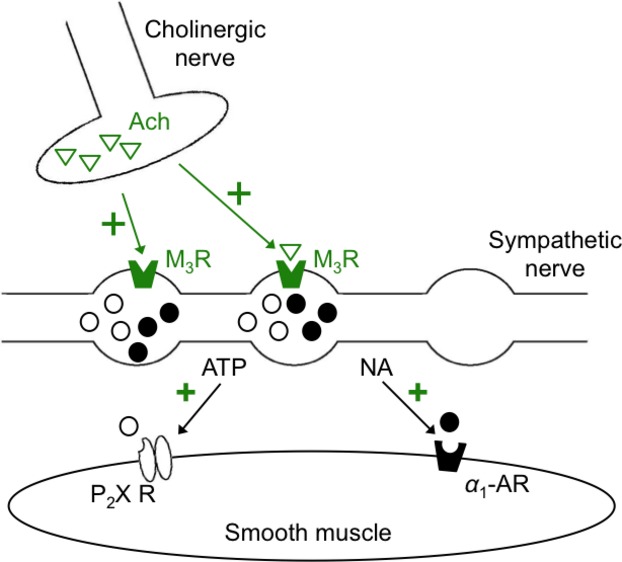
Schematic representation of the excitatory effect of Ach on electric field stimulation-evoked contractions of the rabbit vas deferens. Release of Ach from cholinergic nerves activates prejunctional M_3_ receptors which potentiates the release of ATP and noradrenaline from sympathetic nerves causing an increase in contraction amplitude. Ach, acetylcholine; ATP, adenosine trisphosphate.

The dramatic inhibitory effects of atropine and 4-DAMP on the sustained phase of contraction were surprising because it has been known for many years that the tonic phase of the biphasic contraction is brought about by activation of *α*_1_-adrenoceptors by NA (Ventura 1998; Burnstock and Verkhratsky 2010; Koslov and Andersson 2013). The simplest explanation for these results was that this phase of contraction was due to direct stimulation of MRs on smooth muscle cells by Ach and therefore that this response was primarily cholinergic in nature rather than adrenergic. However, although a direct cholinergic innervation has been reported in murine vas deferens (Cuprian et al. 2005) it seems unlikely to account for the results of this study, for two main reasons. Firstly exogenous application of Cch failed to evoke a response in isolated VDSMC and secondly, we found that both prazosin and guanethidine inhibited the sustained phase of EFS-evoked contractions. These data support the view that the sustained phase of contraction is mediated by NA acting on postjunctional *α*_1_-adrenoceptors (as reported by Sneddon et al. 1984) and that Ach augmented this response by binding to prejunctional M_3_ receptors (M_3_Rs) (Fig. 8). Yet, if this were the case, it was surprising that there was not a residual contraction remaining in the presence of the MR antagonists that would be expected to result from direct stimulation of sympathetic nerves. One possible explanation for the lack of such a response is that EFS may also stimulate an inhibitory pathway that opposes contraction, thus limiting the contraction that would be expected to arise through direct stimulation of sympathetic nerves. In this model the amplitude of EFS-evoked contractions of the vas deferens would not only be determined by direct release of neurotransmitters from sympathetic nerves, but also by activation of cholinergic nerves that enhance contraction and an inhibitory pathway that would reduce contraction.

The possibility that neurogenic contractions of the vas deferens were modulated by an inhibitory pathway was investigated by examining the effects of ODQ, an inhibitor of sGC, on EFS-evoked contractions. sGC is an intracellular enzyme that is responsible for mediating the effects of NO and carbon monoxide (CO) by stimulating production of guanosine 3′,5′-cyclic monophosphate (Friebe et al. 1996; Friebe and Koesling 2003). We reasoned that if such an inhibitory pathway were present then the amplitude of EFS-evoked contractions should increase, when it was inhibited by ODQ. This was indeed the case and, furthermore, the inhibitory effects of 4-DAMP were also reduced in the presence of ODQ, unmasking a contraction that may be due to direct stimulation of sympathetic nerves. Therefore, it appears that the inhibitory effects of 4-DAMP and atropine on EFS-induced contractions may appear exaggerated due to the presence of an, ODQ-sensitive, inhibitory pathway that offsets the expected contraction in response to direct stimulation of sympathetic nerves. These data raise questions about the involvement of NO in this response and if so, whether it acts prejunctionally by preventing release of NA from sympathetic nerves, or postjuctionally, activating sGC in smooth muscle cells to oppose contraction. The precise role of NO in the vas deferens is still unclear. For example, some investigators found that both the purinergic and adrenergic components of EFS-evoked contractions of rat vas deferens were enhanced by the NO synthase inhibitor l-NAME, indicating an inhibitory role for NO in the EFS response (Gur et al. 2010; da Silva et al. 2012). In contrast, (Postorino et al. 1998; Nakanishi et al. 2006) reported that NO facilitates sympathetic neurotransmission in rat and guinea-pig vas deferens rather than inhibiting it. Therefore, it is clear that mechanisms that underlie the effects of sGC in the vas deferens are complex and require further investigation.

The amplitude of EFS-evoked contractions of rabbit vas deferens in this study was potentiated by Ach, Cch, and neostigmine. Similar findings were reported for other species, including rat (Chung and Freer 1983; Lee 1985), guinea-pig (Fukushi and Wakui 1986; Iram and Hoyle 2005), and mouse (Matsuno and Mita 1992) and these effects were attributed to activation of postjunctional Ach receptors. In contrast, Miranda and Wolstenholme (1985) and Miranda et al. (1992) concluded that the enhancement was brought about by activation of prejunctional MRs on sympathetic nerves which enhanced NA release. The precise mechanisms responsible for the stimulatory effects of Ach in this study require further investigation. However, as the potentiation of the EFS-induced contractions by Ach was abolished by both prazosin and guanethidine and since direct application of Cch to freshly isolated VDSMC failed to induce a Ca^2+^ transient or a contraction we speculate that a prejunctional mechanism, involving activation of M_3_Rs, is most likely to account for the results of this study (Fig. 8). However, it should be noted that Iram and Hoyle (2005) reported that potentiation of EFS-evoked contractions of guinea-pig vas deferens were mediated by postjunctional MRs, even though Cch itself did not induce contraction, therefore it is possible that a similar mechanism may account for the results of this study.

A puzzling aspect of this study was that, although the transient element of the EFS-evoked contractions was unaffected by atropine and 4-DAMP, they were potentiated by exogenous application of Ach, Cch, and neostigmine. However, these effects were less consistent than those on the sustained component, as neostigmine did not always induce an enhancement of the transient phase. Furthermore, these effects were also partly reversed by prazosin and guanethidine suggesting that they could be due to release of NA. Similar effects were reported by Miranda and Wolstenholme (1985); who concluded that enhancement of the twitch contraction of rat vas deferens by exogenous application of Ach was not caused by release of endogenous ATP, but involved an effect on presynaptic *α*_2_-adrenoceptors. However, both prazosin and guanethidine have also been shown to reduce release of ATP in guinea-pig vas deferens (von Kügelgen and Starke 1991; Mihaylova-Todorova et al. 2001) therefore it is possible that enhancement of the transient component by Ach could also be due to enhanced release of ATP. It is also possible that the contractility of the vas deferens may be regulated by Ach released from nonneuronal sources, as has been demonstrated in the bladder and the airways, where it has been shown to be released from the urothelium and epithelium, respectively (Wessller and Kirkpatrick 2001; Yoshida et al. 2006).

## Conclusions

This study demonstrates that biphasic neurogenic contractions of rabbit vas deferens smooth muscle are more complex than first thought. The amplitude of EFS-evoked contractions is not just dependent upon direct release of ATP and NA from sympathetic nerves, but results from a balance between activation of M_3_Rs that enhance contraction and an inhibitory sGC-dependent pathway that inhibits contraction.

## Conflict of Interest

None declared.
